# Current and future Disability-Adjusted Life Years (DALYs) of Salmonella and Campylobacter in Belgium

**DOI:** 10.1186/2049-3258-73-S1-K3

**Published:** 2015-09-17

**Authors:** Charline Maertens de Noordhout, Brecht Devleesschauwer, Diallo Lamarana, Juanita Haagsma, Arie Havelaar, Sophie Quoilin, Sophie Bertrand, Yves Dupont, Olivier Vandenberg, Patrick Brandt, Niko Speybroeck

**Affiliations:** 1 UCL, Brussels, Belgium; 2 University of Florida, Gainesville, Florida, United States of America; 3 Université catholique de Louvain, Brussels, Belgium; 4 Erasmus MC, Rotterdam, Nederland; 5 Belgian Scientific Institute of Public Health, Brussels, Belgium; 6 Université libre de Bruxelles, Brussels, Belgium; 7 University of Texas, Dallas, United States of America

## 

Salmonellosis and campylobacteriosis are major foodborne diseases caused by consumption of contaminated raw pork or poultry. Symptomatic disease is mainly characterized by gastroenteritis, although immunoreactive complications also occur, especially among the immunocompromised. Although these diseases cause the highest number of confirmed foodborne bacterial infections in Belgium, their real and future population health impact remains unknown. The objectives of this study were to estimate and forecast the number of *Salmonella* and *Campylobacter* cases in Belgium from 2012 to 2020 and to calculate the corresponding number of Disability-Adjusted Life Years.

The time series of laboratory-confirmed *Salmonella* cases ranged from 2001 to 2012, and that of *Campylobacter* cases from 1993 to 2013. We developed a Bai and Perron two breakpoint model to design salmonellosis time series taking into account the multiple changes in *Salmonella* spp. transmission and control. We developed a dynamic linear model for campylobacteriosis because the data showed a stochastic drift, varied locally and suffered from parameter stability issues around the seasonal component of the data. We calculated DALYs using standard formulas.

The salmonellosis forecast showed a continued decline after 2005. The average monthly number of salmonellosis cases was 264 in 2012 and predicted to be 212 in 2020 (Standard Deviation [SD] 87). Salmonellosis caused 173 DALYs (95% Uncertainty Interval [UI] 33-433) per 100,000 in 2012 and 177 DALYs (95% UI 32-430) per 100,000 in 2020. The average monthly number of campylobacteriosis cases was 633 in 2012, with the predictions showing an upward trend until 2020 to an average of 1081 (SD 311) cases per month. Campylobacteriosis caused 25 DALYs (95% UI 9-60) per 100,000 in 2012 and 40 DALYs (95% UI 14-101) per 100,000 in 2020.

Assuming a constant environment, the burden of salmonellosis will stay stable and the burden of campylobacteriosis may almost double until 2020.

